# Automatic identification of benign pigmented skin lesions from clinical images using deep convolutional neural network

**DOI:** 10.1186/s12896-022-00755-5

**Published:** 2022-10-10

**Authors:** Hui Ding, Eejia Zhang, Fumin Fang, Xing Liu, Huiying Zheng, Hedan Yang, Yiping Ge, Yin Yang, Tong Lin

**Affiliations:** 1 Department of Cosmetic Laser Surgery, Hospital for Skin Disease and Institute of Dermatology, Peking Union Medical College and Chinese Academy of Medical Sciences (CAMS), Nanjing, 210042 China; 2 Jiangsu Key Laboratory of Molecular Biology for Skin Diseases and STIs, Institute of Dermatology, Chinese Academy of Medical Sciences and Peking Union Medical College, Nanjing, 210042 China

**Keywords:** Artificial intelligence, Image processing, Computer-assisted, Deep learning, Patient identification systems, Pigmentation disorders

## Abstract

**Objective:**

We aimed to develop a computer-aided detection (CAD) system for accurate identification of benign pigmented skin lesions (PSLs) from images captured using a digital camera or a smart phone.

**Methods:**

We collected a total of 12,836 clinical images which had been classified and location-labeled for training and validating. Four models were developed and validated; you only look once, v4 (YOLOv4), you only look once, v5 (YOLOv5), single shot multibox detector (SSD) and faster region-based convolutional neural networks (Faster R-CNN). The performance of the models was compared with three trained dermatologists, respectively. The accuracy of the best model was further tested and validated using smartphone-captured images.

**Results:**

The accuracies of YOLOv4, YOLOv5, SSD and Faster R-CNN were 0.891, 0.929, 0.852 and 0.874, respectively. The precision, sensitivity and specificity of YOLOv5 (the best model) were 0.956, 0.962 and 0.952, respectively. The accuracy of YOLOv5 model for images captured using a smart-phone was 0.905. The CAD based YOLOv5 system can potentially be used in clinical identification of PSLs.

**Conclusion:**

We developed and validated a CAD system for automatic identification of benign PSLs using digital images. This approach may be used by non-dermatologists to easily diagnose by taking a photo of skin lesion and guide on management of PSLs.

## Introduction

Benign pigmented skin lesions (PSLs) are caused by either over production of melanin or abnormal increase in the density of active melanocytes [1]. These changes may result in severe skin disfigurement, affecting the mental health and quality of life of the patient [2]. Common benign PSLs mainly include freckles, melasma and nevus of Ota [3]. Asians, in particular, show a greater tendency to develop skin pigmentation disorders, which requires special clinical management and selective use of cosmetics [4]. However, most skin disease or diagnoses are performed by non-specialists, in particular, general physicians. Although all PSLs result from abnormalities in melanocytes and exhibit almost similar clinical manifestations, the treatment methods and prognoses of various sub-types may be quite different. Misdiagnosis can worsen the clinical outcomes by delaying the appropriate treatment. This underscores the need for accurate methods in identification of benign PSLs.

In recent years, with the development of machine learning in the field of computer vision, medical image processing research based on machine learning has become a hot research topic in the field of computer-aided diagnosis. There are numerous computer-based systems in the fields of ophthalmology, oncology and dermatology that utilize digital images in disease diagnosis [5–7]. The realization of image classification by machine learning provides a lot of reference content for the classification of clinical images of skin diseases. As AlexNet won the championship in ImageNet image recognition contest in 2012 with excellent performance, deep learning machine vision algorithm based on convolutional neural network has attracted more and more attention from researchers, and has rapidly become the mainstream method in image classification, target detection and image segmentation. Foreign studies of dermatological pathological maps were relatively early. Cascinelli et al. [8] proposed an automatic classification algorithm study of pigmented skin lesions (PSL) images. Umbaugh et al. [9] proposed the feature extraction method of color moments and image segmentation method to diagnose skin cancer. Stanganelli et al. [10] used fluorescence microscope images and support vector machine classifier to classify dermatological case maps. Kassem et al. [11] applied the transfer learning to the Alex-net in different ways to classify the skin lesions into different classes. They [12] also proposed a novel method for seven kinds of skin lesion classification based on AlexNet and the performance of model exceeds other classification method by at least 6%. Esteva et al. [13] paper published in Nature used deep learning convolutional neural network to detect melanoma from a dermatoscopic image dataset, with an accuracy of 94%. However, the author used a very large dataset containing 120,000 dermatoscopic images, and the dataset was not published. Image pretreatment, image segmentation, image feature selection and feature extraction, and image classification and recognition are the main directions of current dermatological case map research, but most of the studies are based on dermatoscopic images, and there are few studies based on clinical dermatological images. However, the availability of dermoscope is mostly limited to dermatologists, especially those in rural area. And dermoscopes are unnecessary for the diagnosis of many common skin diseases. In contrast, given the availability of digital images, most recent studies have explored the possibility of identifying skin lesions using digital images.

Based on our task of detecting and classifying skin diseases, we need to use object detection algorithm. Object detection is one of the basic tasks in the field of computer vision, and has been studied in academia for nearly two decades. In recent years, with the rapid development of deep learning technology, the target detection algorithm has also shifted from the traditional algorithm based on manual features to the detection technology based on deep neural network. From the original R-CNN [14] and OverFeat [15] proposed in 2013, to the Fast/Faster R-CNN [16, 17], SSD [18], YOLO [19] series, and to the recent Pelee [20] in 2018. In less than five years, target detection technology based on deep learning has emerged a lot of algorithms with good performance, realizing the structure from two stages to one stage, from bottom-up only to top-down, from single scale network to feature Pyramid Network. These algorithms have excellent detection effect and performance on open target detection dataset.

Herein, we generated a large, dataset of facial clinical images for six benign PSLs. We then developed a deep convolutional neural network (DCNN) for accurate identification and classification of characteristic disease lesions for 6 benign PSLs. The model was validated using several tests. For instance, the findings of the model were compared with reference results generated by experts. Because phones are more readily available, we further compared between digital camera and smart phone generated images in identifying PSLs.

## Material and methods

### Datasets

The protocols for this study were approved by the Ethics Committee of Chinese Academy of Medical Sciences (CAMS), Hospital for Skin Diseases. We captured 12, 836 facial clinical images of six most common PSLs from patients seeking treatment at the Institute of Dermatology, Chinese Academy of Medical Sciences and Peking Union Medical College between 2004 and 2019. There were 2557 Fitzpatrick skin type III and 10,279 Fitzpatrick skin type IV cases. Patients also presented with all the six common benign PSLs; lentigines, freckles, melasma, café-au-lait spots, nevus of Ota and Hori's nevus. All images for the skin lesions were captured using digital single lens reflex (DSLR) cameras (EOS 550D/800D, Canon or FinePix S9500, Fujifilm cameras, all from Japan) or smartphone cameras.

We only used images clinically diagnosed and classified into either of the six diseases types. The process was performed independently by 3 dermatologists. For practical cases, images from one patient can sometimes simultaneously present multiple types of diseases, such images are also included in the computing dataset. Blurred images or of atypical lesions were excluded.

The annotation of images and the calibration of diagnostic model were performed by three experienced dermatologists. The classified images were further reviewed by two other experienced senior dermatologists. The lesions were labeled (available at https://github.com/tzutalin/labelImg) using a rectangular mark(s), abbreviated as sl (solar lentigines), fre (freckles), mel (melasma), caf (café-au-lait spots), ota (nevus of ota) and hori (Hori's nevus).

Overall, there were 12,836 images, which were divided in to training (n = 10,269; 80%) and validation set (n = 2567; 20%). Two test sets were used to evaluate the performance of the models. One data set comprised of 300 unique but randomly selected pictures, captured by DSLR cameras. The second control set (PDCset) consisted of 50 pictures. All the six disease types were represented in the 300 picture set, almost in equal proportion. The third set consisted of 120 images, with equal proportion of the 6 diseases types. For this dataset, 20 control images (PHCset) captured using smartphone cameras were used. The images never overlapped among the four datasets (Table 1).

**Table 1:** Various network model versus dermatologist’s precision, sensitivity, and specificity values

Diseases	SSD			Faster R-CNN			YOLOv4			YOLOv5			DOCTOR		
	Precision	Sensitivity	Specificity	Precision	Sensitivity	Specificity	Precision	Sensitivity	Specificity	Precision	Sensitivity	Specificity	Precision	Sensitivity	Specificity
Sl	0.938	0.882	0.940	0.955	0.840	0.961	0.920	0.92	0.922	0.920	0.92	0.922	1.000	0.967	1.00
Fre	0.960	0.923	0.960	0.909	0.980	0.902	0.959	0.904	0.96	1.000	0.942	1.00	0.994	0.994	0.980
Mel	0.850	0.927	0.836	0.877	0.862	0.865	0.877	0.877	0.870	0.930	0.93	0.925	0.978	0.989	0.926
Caf	0.882	0.94	0.887	0.980	0.990	0.941	0.927	1.00	0.922	0.944	1.00	0.922	1.000	0.993	1.00
Ota	0.979	1.00	0.980	0.978	0.938	0.980	0.960	1.00	0.961	0.980	0.98	0.980	1.000	1.00	1.00
Hori	0.906	0.980	0.906	0.940	0.940	0.942	0.958	0.958	0.961	0.962	1.00	0.962	0.993	0.981	0.980
Average	0.919	0.942	0.918	0.940	0.925	0.932	0.934	0.943	0.933	0.956	0.962	0.952	0.994	0.987	0.981

Considering that annotation procedure of the training dataset (i.e., annotate the location and the region of the lesions on the clinical images) is beyond the capability of manual work, an automatic annotating method was employed to pre-process the dataset. We first manually labeled 4868 images, which was then used to derive a methodical model that can automatically annotate the rest 7968 images. After verification, the annotated images were incorporated in the training dataset to strengthen the credibility of results derived from the set and reduce on labeling constraints.

### Deep convolution neural networks (DCNNs) training

We selected YOLO V4, YOLO V5, SSD, Faster RCNN as the experimental network among the current popular detection networks. Detection algorithms can be divided into two categories according to calculation steps, one is the one stage algorithm and the other is the two stage algorithm. The main difference between the two is that the two stage algorithm requires a proposal (a pre-selection box that may contain the object to be detected), and then carries out fine-grained object detection. The one stage algorithm extracts features directly in the network to predict object classification and location.

### Deep convolution neural networks (DCNNs) technology

The basic idea of YOLO algorithm is to use end-to-end convolutional neural network to directly predict the category and location of the target. The YOLO algorithm divides the input image into S × S grid cells, and each grid is responsible for predicting the target whose center point falls within the grid. In the image labeling stage, the label category of the grid where the target center point is located is the target category, and the label category of other grids is the background.

Each grid predicts C categories, where C represents the number of target categories plus the number of background categories. In addition, each grid will predict B boundary boxes, which will share the predicted scores of C categories. The predicted value of each bounding box includes position, size and Confidence, that is, x, y, W, H, Confidence. X and y are the center coordinates of the boundary box. This coordinate belongs to the relative coordinate and represents the offset of the center point of the boundary box relative to the upper left corner of the grid. W and H also represent the relative size, representing the scaling value of the edge length of the boundary box relative to the edge length of the original picture. In order to eliminate the influence of difference in loss contribution of targets with different sizes, square root calculation is used for loss calculation of W and H. Confidence is a prediction parameter unique to YOLO models. It represents the product of prediction frame, real frame IOU and the probability of existence of target. Therefore, it contains both the Confidence of existence of target and the coincidence degree of prediction and real frame. Formula is as follows.

1 $$Conf = {\text{Pr}}\left( {Object} \right) \times IOU_{pred}^{truth}$$

YOLOv5 model uses adaptive prior frame algorithm: K-means clustering algorithm and genetic learning algorithm are used to cluster and learn all target boundary frames in the data set to generate the prior frame size with the highest matching degree with the data set, so as to avoid the error caused by manual design of prior frame size.

The network structure of YOLOv5 model adds Focus module between input and feature extractor, and also uses CSPDark Net53 module, SPP module and PANet module in other places.

The loss function of YOLOv5 model includes confidence loss function, class loss function and position loss function.

The confidence loss function is expressed as follows.

$$\mathop c\limits^{ \wedge }{_{i}} = Sigmoid\left( {c_{i} } \right)$$

2 $${\text{L}}_{{{\text{conf}}}} \left( {\text{o,c}} \right) = \frac{{\sum\nolimits_{i} {\left( {o_{i} \ln \left( {\mathop {c_{i} }\limits^{ \wedge } } \right) + \left( {1 - o_{i} } \right){\text{ln}}\left( {1 - \mathop {c_{i} }\limits^{ \wedge } } \right)} \right)} }}{{\text{N}}}$$

where ${\text{O}} \in \left\{ 0, 1 \right\}$: true confidence value, when sample i is a positive sample, is 1; otherwise, it is 0; $\mathop {\text{C}}\limits^{ \wedge }{_{{{\text{ij}}}}}$: Prediction confidence value, indicating that model prediction sample I is positive sample probability; N: total number of positive and negative samples.

The category loss function is formulated as follows.

$$\mathop {\text{C}}\limits^{ \wedge }{_{ij}} = Sigmoid\left( {{\text{C}}_{ij} } \right)$$

3 $$L_{da} \left( {O,C} \right) = \frac{{\sum\limits_{i \in pos} {\begin{array}{*{20}c} {} \\ {} \\ \end{array} \sum\limits_{j \in cla} {\left( {O_{ij} {\text{ln}}\left( {\mathop {C_{ij} }\limits^{ \wedge } } \right) + \left( {1 - O_{ij} } \right){\text{ln}}\left( {1 - \mathop {C_{ij} }\limits^{ \wedge } } \right)} \right)} } }}{{N_{pos} }}$$

where ${\text{O}}_{{{\text{ij}}}} \in \left\{ {0}, {1} \right\}$: true category value, if there is a class j target in the sample i, the value is 1; otherwise, the value is 0; $\mathop {\text{C}}\limits^{ \wedge }_{{{\text{ij}}}}$: Prediction category value, indicating the existence of the probability of class j targets in the model prediction sample i; ${\text{N}}_{{{\text{pos}}}}$: Positive sample number.

The locating loss function is formulated as follows.

4 $$L_{{loc}} \left( {I,g} \right) = \frac{{\sum\limits_{{i \in pos}} {\sum\limits_{{m \in \left\{ {x,y,w,h} \right\}}} {L_{{GIoU}} \left( {\hat{I}_{i}^{m} - \hat{g}_{i}^{m} } \right)} } }}{{N_{{pos}} }}$$

The YOLOv5 model uses the GIOU loss function to calculate the model positioning loss. Compared with the error square sum loss function, the GIOU loss function can better reflect the degree of coincidence between two rectangular frames, and has scale invariance. It also solves the situation that when two rectangular frames do not intersect, the formula of IOU loss function is − ln0, which has no mathematical significance.

GIOU loss function formula is as follows.

$$IoU = \frac{area\left( C \right) \cap area\left( G \right)}{{area\left( C \right) \cup area\left( G \right)}}$$

5 $$GIoU = {\text{I}}oU - \frac{{A_{c} - u}}{{A_{c} }}\begin{array}{*{20}c} {} & {} \\ \end{array} \left( { - 1 \le GIoU \le 1} \right)$$

$$L_{GIoU} = 1 - GIoU\begin{array}{*{20}c} {} & {} \\ \end{array} \left( {0 \le L_{GIoU} \le 2} \right)$$

In addition, we propose an improved approach based on the YOLOv5 model. The loss function of YOLOv5 model is obtained by calculating the sum of the bounding box regression loss, class loss, and confidence loss:

6 $${\text{Loss}} =\uplambda _{{{\text{box}}}} *{\text{loss}}_{{{\text{box}}}} +\uplambda _{{{\text{obj}}}} \;{\text{loss}}_{{{\text{obj}}}} +\uplambda _{{{\text{cls}}}} \;{\text{loss}}_{{{\text{cls}}}}$$

where λ_box_, λ_obj_ and λ_cls_ are the corresponding weighting factors. Here, the bounding box loss (loss_box_) uses CIoU, the class loss (loss_cls_) is calculated through BCE (Binary Cross Entropy) loss, and the confidence loss (loss_obj_) is realized by BCE with logits loss to get numericalstability. The difference is that the losscls is calculated by:

7 $${\text{loss}}_{{{\text{cls}}}} \left( {p,p*} \right) = - \left( {p\log \left( {p*} \right) + \left( {1 - p} \right)\log \left( {1 - p*} \right)} \right)$$

where p and p* are the groudtruth and predicted values of the categories, respectively. In particular, if anchors overlap the target bounding box and the IOU is greater than 0.5, they are considered as positive labels (p* = 1), and if the overlap area is less than 0.02, they are considered as negative labels (p* = 0). In this paper, the improved YOLOv5 network was trained by stochastic gradient descent (SGD) in an end-to-end way. The batch size of the model training was set to 8, and learning rate was 0.002. The momentum factor (momentum) was set to 0.9, and the decay rate (decay) of weight was set to 0.0005.

**Fig. 1:**
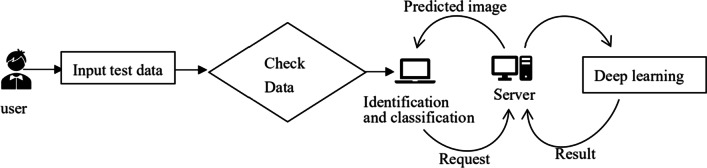
Schematic process of automatic identification of benign pigmentary lesions

We use these four models to train and test our data sets respectively. To ensure the effectiveness of the model, we first use ImageNet to pre-train the model, and then use our face data set for training. The training and verification process of the model is shown in Fig. 1. For the experimental hardware part, an NVIDIA GeForce RTX 1080 was used for training.

### Statistical analysis and evaluation of the AI model

The performances of the four deep learning algorithms were assessed based on their precision, sensitivity, specificity and accuracy in diagnosing the PSLs. These measures are computed using the following equations:

8 $${\text{Precision}} = \frac{TP}{{TP + FP}}{ }$$

9 $${\text{Sensitivity}} = \frac{TP}{{TP + FN}}$$

10 $${\text{Specificity}} = \frac{TN}{{FP + FN}}$$

11 $$Accurancy = \frac{TP + TN}{{TP + FP + FN + TN}}$$

where TP, FP, FN, and TN refer to true positive, false positive, false negative, and true negative respectively.

The Confusion matrix.

The mean average precision (mAP) is the index to evaluate the quality of the model in target detection task, that is, the Average value of AP of various categories. AP is a comprehensive measure of accuracy and recall. Accuracy rate and recall rate in target detection can be commonly understood as precision rate and recall rate.

The formulas for precision and recall are as follows.

12 $$\begin{gathered} Precision = \frac{TP}{{\left( {TP + FP} \right)}} \hfill \\ Recall = \frac{TP}{{\left( {TP + FN} \right)}} \hfill \\ \end{gathered}$$

To maintain consistency with the clinical diagnosis process, the neural network was tailored such that it can simultaneously yield the diagnose results for more than one disease subtypes. Further, during the calculation of the neural network’s accuracy, we regard the diagnose result of a certain test sample correct, only when all subtypes are simultaneously correctly diagnosed. The efficacy of the models was compared by two dermatologists double-blinded to the study. All statistical analyses were performed using SPSS V. 23 (IBM, Chicago, IL, USA). *P* value < 0.05 was considered statistically significant.

## Results

For the training set, we analyzed 12,836 images, including 2232 for solar lentigines, 2239 for freckles, 2268 for melasma, 2267 for café-au-lait spots, 2236 for nevus of Ota and 2239 for Hori’s nevus. The proportion of images for each disease in the training, validation and testing sets were generally equal to ensure balanced representation.

**Fig. 2:**
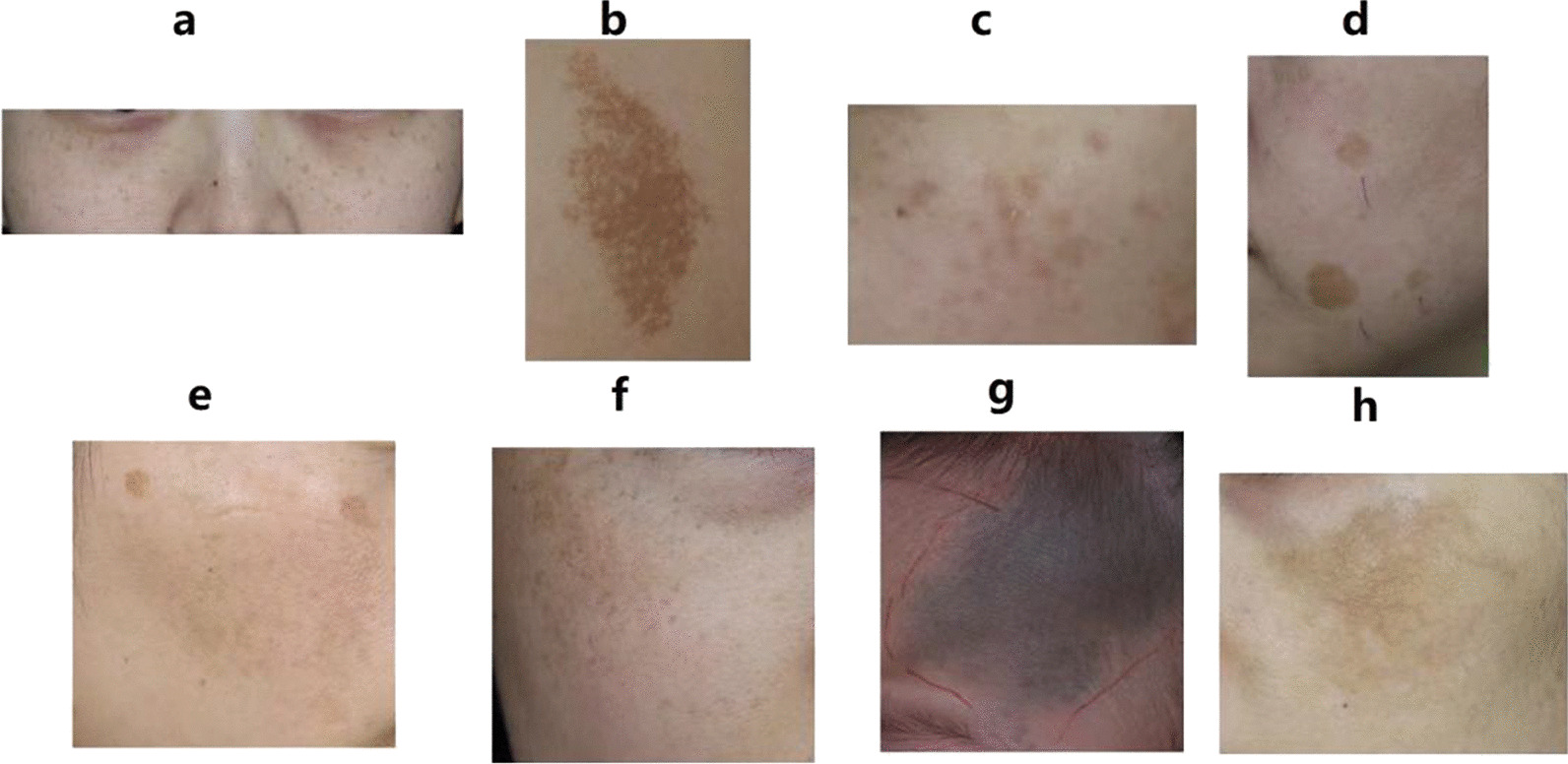
Representative identification results for single disease and the coexistence of multiple diseases achieved by DCNN. **a** freckles, **b** café-au-lait spots, **c** Hori's nevus, **d** solar lentigines, **e** solar lentigines and melisma, **f** freckles and melisma, **g** nevus of Ota, **h** melisma. The white rectangular paper on the patients’ face is the case number listed according to the patient's disease and the order of visit. But it is not related to the training of our model

**Fig. 3:**
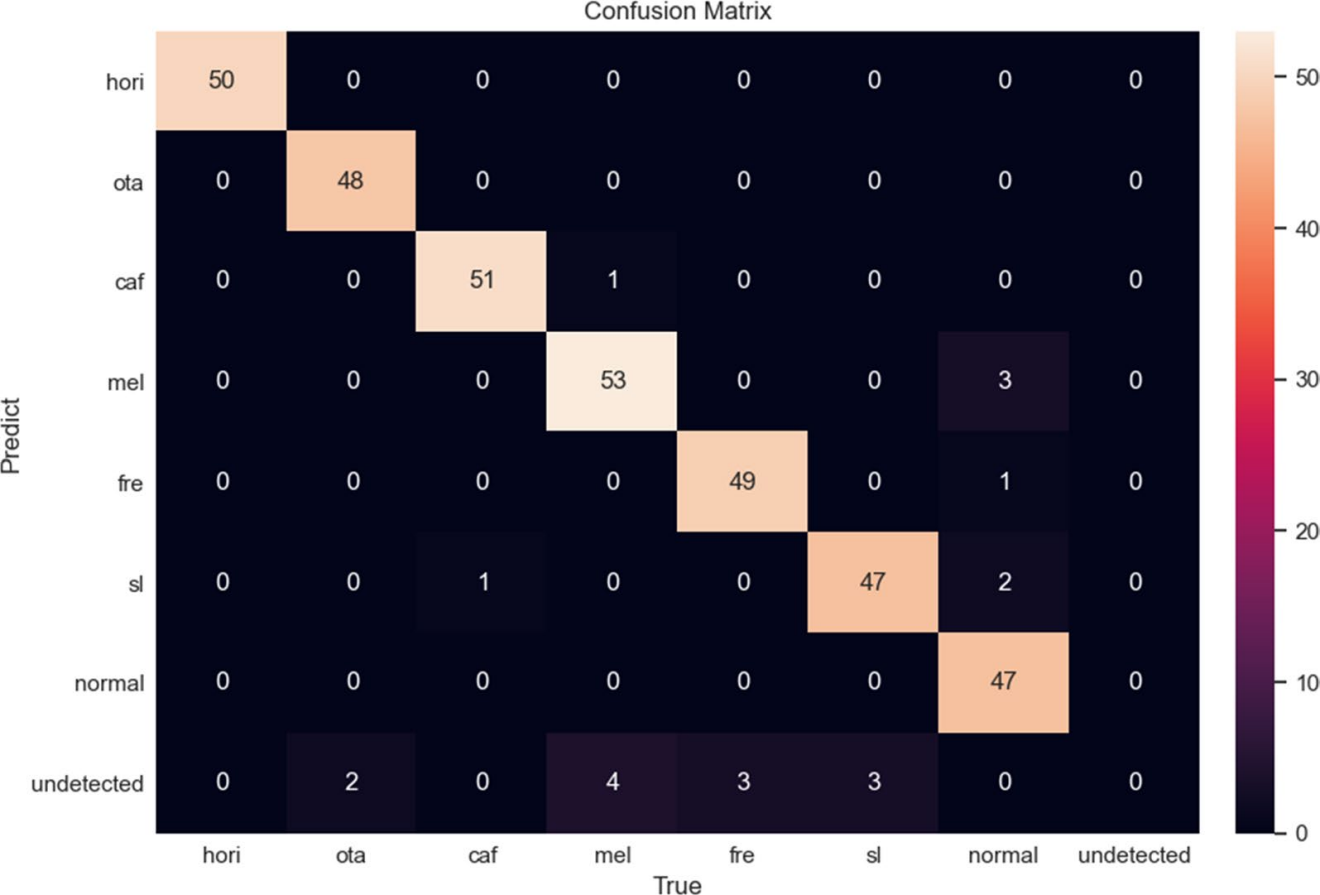
Confusion matrix

The DCNN was used to assess the predictive value of the model. The process of identifying the six benign PSLs using the PDCset is shown in Fig. 2. We compare the performance measure with state-of-art that works on the same dataset, PDCset. The accuracies of YOLOv4, YOLOv5, SSD and Faster R-CNN model were 0.891, 0.929, 0.852 and 0.874, respectively. For the external validation dataset, the proposed model based YOLOv5 exhibited the best accuracy of 0.929. The performance of the proposed model based YOLOv5 has exceeded the existing classification methods. The precision, sensitivity, and specificity the four models for diagnosing the six benign PSLs is shown in Table 1. The confusion matrix is shown in Fig. 3.

Based on its performance, YOLOv5 model was selected for the diagnosis of PSLs. The accuracy rate of the dermatologists was 0.949. The model's performance of identifying 6 kinds of diseases was similar to that of dermatologists. The precision, sensitivity, and specificity of dermatologists for diagnosing the six benign PSLs are shown in Table 1.

The accuracy of YOLOv5 model for the PHCset was 0.905. YOLOv5 model also has good performance on untrained data sets, which images were taken by smart photos. The precision, sensitivity, and specificity of YOLOv5 model for diagnosing the six benign PSLs are shown in Table 2.

**Table 2:** Precision, sensitivity, and specificity values of YOLOv5 model on PHCset

Diseases	Precision	Sensitivity	Specificity
Sl	0.850	0.810	0.857
Fre	1.000	0.880	1.000
Mel	0.760	0.826	0.740
Caf	0.905	0.950	0.900
Ota	1.000	1.000	1.000
Hori	1.000	0.850	1.000
Average	0.919	0.886	0.916

## Discussion

Deep learning has emerged as a promising tool for diseases, including dermatological complications. The majority of previous studies have focused on melanoma, skin inflammatory diseases and other skin cutaneous tumors [21–24]. Meanwhile, dermatological models can be used in the diagnosis and classification of skin lesions. Visual identification of skin complications is largely based on dermoscopic, ultrasound or pathological images, which require special equipment to generate and may not be applicable for certain diseases [25]. In several instances, clinical images can accurately and easily be used in disease diagnosis. However, only few studies have explored the utility of deep learning in the diagnosis of benign PSLs using images captured by smartphones. In this study, we evaluated the application of CAD system for automatic identification of facial benign PSLs. The CAD utilizes the deep learning technology, in which millions of images for natural diseases are analyzed to develop the identification model. The precision, sensitivity and specificity of the four models against the six PSLs were above 0.950, close to the diagnostic standards by qualified dermatologists. The accuracy of YOLOv5 model for the PHCset was 0.905, whereas its average sensitivity, specificity and precision were 0.886, 0.916 and 0.919, respectively. This demonstrates the diagnostic potential of the model identifying skin complications using digital photos. The close match of the diagnose results, in terms of the average sensitivity, specificity and precision, between testing images taken by different types of cameras in different photographic scenes, demonstrating that our automatic diagnosis model has a high tolerance to the photographic conditions such as the camera, ambient light, focal length and angle.

Besides, YOLOv5 model could accurately detect different PSLs in the same photograph, contrary to a previous model which could only analyze images with one lesion image [26]. Accurate identification of skin lesion has a critical clinical significance. For example, even though melasma and Hori's nevus are almost alike, they require distinct treatment.

At the moment, the performance of deep learning based models heavily relies on the quality and quantity of data. For our model, no substantial preprocessing of the images such as cutting off certain sections of the images, usually performed to improve the quality of the images, was warranted. This was probably because the large group of images used in this study were acquired at a basically fixed setting. In addition, the quality of the images was almost the same, and differences in illumination across over time were already resolved by a deep learning based approach, which minimized possible sources of errors arising from varied resolutions.

Even though the use 12,836 images in the training and validating set enhanced the performance of the eventual models, annotation of the large data was cumbersome. Razeghi [27] demonstrated the application of human in the loop visual recognition activity in developing interactive skin lesion recognition system. The human system significantly enhances the recognition accuracies of images. Meanwhile, the development of our model was inspired by this method. The performance of the model was validated by experienced dermatologists. A small set of manually labeled images can be used for subsequent automatic labeling, to generate annotated files. After professional review, the annotated images are incorporated with training dataset to increase the amount of data. In addition, any errors by model can easily identified and corrected promptly. Overall, the models set the foundation for automatic annotation of clinical images, thus improving the general efficiency.

The aim of this study was to improve diagnosis of skin complications, particularly by non-skin specialists. The model is also very useful in performing diagnoses in rural areas, where access to specialized treatment is limited. General practitioners can use CAD for accurate diagnosis and differentiation of benign PSLs, reducing the over reliance on dermatologists. Given the intuitive diagnostic features, the CAD model can also be used in training medical students. For patients, early self and accurate diagnosis improves treatment benefits. In addition, the system if flexible and can be customized to soot specific needs of a given institution. Continuous generation of data and derivation of algorithms will only improve the application of deep learning convolutional neural network, which can potentially revolutionize the health care system. In our future study, we shall provide mobile phone application for self-diagnosis, treatment and management of PSLs.

Regarding limitations, first we only focused on six benign PSLs. Several rare skin complications were not included, but may still be clinically important diseases. Also, we only assessed Fitzpatrick skin types III–IV. As such, more studies are needed to incorporate greater range of skin lesions. Third, the data was only generated from a single center, thus may not represent regional variability in characteristic disease presentation. Forth, only facial images were used. The performance of the model for images derived from other areas remains uncertain. Fifth, the images were all derived from China nationals, thus the performance of the model in individual from other ancestry remain to be validated. Also, images captured by phones are of poor resolution, relative to those by DSLR cameras. Lastly, pictures in the training set were relatively few. Overall, more research is needed to validate the utility and performance of our model.

## Conclusion

CAD is a reliable deep learning-based system that can accurately differentiate six benign PSLs using digital images. This system can be used by non-dermatologists to accurately identify the above skin complication by taking a photo of the skin lesion themselves instead of looking for help from a dermatologist, which aids in timely treatment and management of the diseases.

## Data Availability

The datasets generated and/or analysed during the current study are not publicly available but are available from the corresponding author on reasonable request.
